# Getting Closer to Decrypting the Phase Transitions of Bacterial Biomolecules

**DOI:** 10.3390/biom12070907

**Published:** 2022-06-28

**Authors:** Katarzyna Sołtys, Aneta Tarczewska, Dominika Bystranowska, Nikola Sozańska

**Affiliations:** Department of Biochemistry, Molecular Biology and Biotechnology, Faculty of Chemistry, Wroclaw University of Science and Technology, Wybrzeże Wyspiańskiego 27, 50-370 Wroclaw, Poland; aneta.tarczewska@pwr.edu.pl (A.T.); dominika.bystranowska@pwr.edu.pl (D.B.); nikola.sozanska@pwr.edu.pl (N.S.)

**Keywords:** liquid–liquid phase separation, membraneless organelles, phase transitions, biomacromolecular condensates, multivalent interactions, bacterial cells

## Abstract

Liquid–liquid phase separation (LLPS) of biomolecules has emerged as a new paradigm in cell biology, and the process is one proposed mechanism for the formation of membraneless organelles (MLOs). Bacterial cells have only recently drawn strong interest in terms of studies on both liquid-to-liquid and liquid-to-solid phase transitions. It seems that these processes drive the formation of prokaryotic cellular condensates that resemble eukaryotic MLOs. In this review, we present an overview of the key microbial biomolecules that undergo LLPS, as well as the formation and organization of biomacromolecular condensates within the intracellular space. We also discuss the current challenges in investigating bacterial biomacromolecular condensates. Additionally, we highlight a summary of recent knowledge about the participation of bacterial biomolecules in a phase transition and provide some new in silico analyses that can be helpful for further investigations.

## 1. Introduction

Remarkable diversity among bacterial genomes has been demonstrated many times through genome sequencing analyses [1]. However, compared with eukaryotic genomes, bacterial genomes appeared to be shorter and represented by an unpretentious smaller number of genes and hence a smaller number of proteins. Therefore, it might be difficult to believe that microbes have adapted to their environments, even when using only a limited number of genes and their products. In addition, one of the central biochemical paradigms shifted, and instead of being focused on one particular protein, the paradigm started concentrating on multiprotein complexes by which distinct cellular functions could be executed. Decades of research into biochemistry, structural biology, and biophysics have produced a remarkable compendium of knowledge on the function and molecular properties of individual proteins. The conclusion was clear that proteins rarely act alone. There are many advantages of organizing proteins into higher-order assemblies by facilitating the coordination of cellular functions, increasing the rate of complex reactions, or enhancing the potential for their regulation [2]. The number of possible functional states increases significantly as a function of growing protein–protein interaction networks, and this is especially important for cells that lack intracellular compartments. Therefore, this additional piece of the puzzle, which involves intracellular organization, also seems to be a critical element for small cells that perform complex biological functions. A new aspect of the cellular organization is the membraneless organelles (MLOs) [3], which have been extensively characterized in eukaryotic cells but have been neglected for a long time regarding the function of prokaryotic cells. MLOs are formed via a spontaneous process that is termed liquid–liquid phase separation (LLPS) [4]. LLPS can be driven by a variety of weak, multivalent interactions between biomolecules (e.g., proteins and nucleic acids) [5]. Proteins that contain intrinsically disordered regions (IDRs) or low complexity regions (LCRs) play an important role in the formation of MLOs [6]. Biomacromolecular condensates contain many different components, for which a particular role can be assigned [7]. Protein components can be classified into two main groups. Scaffold proteins are essential constituents of each condensate and are responsible for its integrity and clients, which reside in the MLOs only under certain conditions. MLOs usually possess liquid-like properties [3]. However, different factors (e.g., the concentration of proteins, the presence of nucleic acids, posttranslational modifications, temperature, pH, and salt concentration) can influence their properties and organization [8]. The protein assemblies might, therefore, display a spectrum of material properties, from highly dynamic liquid droplets to solid-like amyloid fibers [9]. The function of biomacromolecular condensates is correlated with their physical state. They mediate stress responses, provide efficient means for the transport and sorting of proteins and metabolites, and can accelerate the assembly of metabolic and signaling complexes [10,11].

This review focuses on structures in bacterial cells that resemble eukaryotic MLOs. We describe bacterial biomacromolecular assemblies for which LLPS propensity was observed, and, together with experimental methods, condensate formation is investigated. Additionally, we provide some bioinformatic analysis of the in silico methods commonly used to evaluate the potential of proteins that undergo LLPS. As there are numerous important questions that remain unanswered regarding this topic, the presented work opens up the conversation and discusses the significance of phase separation in bacteria. We expect that the set of data described here will almost surely be extended in the near future with new examples of proteins that have a propensity to undergo LLPS and new methods of their investigation.

## 2. Bacterial Proteins with a Potential Propensity for Liquid–Liquid Phase Separation

**Figure 1:**
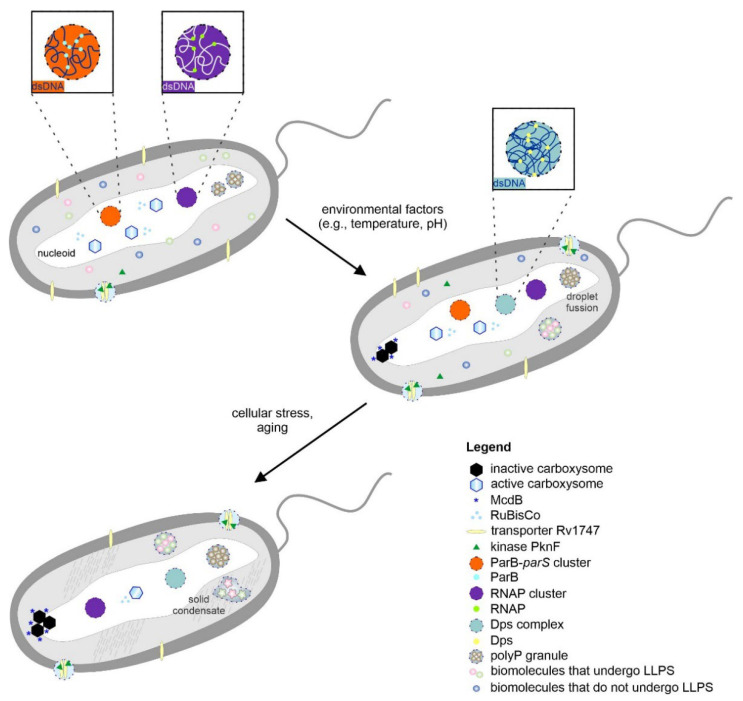
Proposed phase transitions in bacterial cells. Bacterial biomacromolecular assemblies can be present in optimal growth conditions (e.g., bacterial microcompartments). The condensates can fuse upon contact, and the components can be exchanged between condensates. Sudden changes in the environment (e.g., stress) can lead to phase transitions to form more solid-like forms. The formation of such condensates may provide an efficient means for protecting genetic material. For more details, see the main text.

In contrast to eukaryotic proteins, the localization of bacterial proteins is not limited to specific subcellular compartments that are restricted by membrane-surrounded organelles (Figure 1). This is still a complex environment in which some of the proteins diffuse freely in the cytoplasm, while others are found to be a part of an intricate subcellular organization embedded in its local microenvironment [12]. The formation of liquid-phase macromolecular condensates of bacterial proteins was unwittingly suggested and characterized (although not directly) over 20 years ago, when the bacterial nucleoid was hypothesized to be a liquid phase [13]. Currently, the number of scientific papers describing the process of liquid-phase condensation of prokaryotic proteins is growing faster than ever. Below, we discuss some of the most distinctive examples. These bacterial proteins were found to be engaged in important cellular processes, such as cell division with the formation of the partition complex itself, the assembly of clusters of RNA polymerases, or the regulation of stress response pathways.

### 2.1. The Dynamic and Adaptable Nature of Bacterial Carboxysomes May Indicate Their Liquid-Like Character

Bacterial microcompartments (BMCs) are considered to be a class of self-assembling supramolecular structures and are found in approximately 17% of bacterial species [14]. Unlike eukaryotic organelles, BMCs are formed entirely of proteins (10–20 of different types) and provide selectively permeable compartments. The function of BMCs is dedicated to encapsulating selective metabolic enzyme molecules to protect them from competing reagents/reactions or to protect the cell from cytotoxic metabolic intermediates [15]. BMCs are classified into the following major categories: carboxysomes (which are involved in anabolic processes) and metabolosomes (which are involved in catabolic processes). Carboxysomes are found in all cyanobacteria and in some chemoautotrophic bacteria and encapsulate carbonic anhydrase and ribulose-1,5-bisphosphate carboxylase/oxygenase (RuBisCo), which has been claimed to be the most abundant protein on Earth [16]. Both colocalized enzymes function to fix CO_2_ as part of the Calvin–Benson–Bassham cycle and prevent the leakage of CO_2_ into the cytosol. Carboxysomes can be divided into two subtypes (α and β), depending on their protein composition, including the form of RuBisCo that they enclose. In contrast, metabolosomes are functionally more diverse. They are involved in the degradation of different carbon sources, such as 1,2-propanediol [17], ethanolamine [18], fucose and rhamnose [19], or choline [20]. A more detailed review of BMCs’ structural and functional diversity can be found elsewhere [15,21]. Due to their permeable protein shell, carboxysomes are not considered typical MLOs. Despite this, their main composing proteins were observed to form liquid droplets in vitro. RuBisCo of α-carboxysomes was observed to form droplets upon interaction with the intrinsically disordered N-terminal domain of the CsoS2 protein [22], whereas RuBisCo of β-carboxysomes was shown to undergo LLPS as a result of the interaction with the short form of the scaffold protein CcmM, M35 [23]. Additionally, fluorescence recovery after photobleaching (FRAP) measurements of droplets composed of RuBisCo-M35 exhibited a slower rate of recovery when M35 was in its reduced form than with oxidized M35, suggesting that the RuBisCo-M35 condensate was more dynamic under oxidizing conditions, which is the anticipated internal microenvironment of the mature carboxysome [24]. Further analyses by MacCready et al. in the cyanobacteria *Synechococcus elongatus* identified that a novel intrinsically disordered protein (IDP), McdB, interacts with carboxysome shell proteins. McdB was found to undergo phase separation in vitro [25]. Although the factors that regulate the LLPS activity of McdB, as well as the regions of this uncharacterized protein required for such activity, remain to be elucidated, it was observed that McdB forms liquid condensates in a pH-dependent manner [25]. Such a preference may suggest that the acidic nature of the carboxysome, relative to the cytosol of *S. elongatus* at pH 8 [26], may facilitate the LLPS of McdB in the vicinity of carboxysomes [25]. Taken together, all the abovementioned studies suggest that LLPS might be a common feature that characterizes the interactions between RuBisCo and its IDP partners (Figure 1).

### 2.2. Nanoclustering of the Bacterial ATP-Binding Cassette Transporter Rv1747

**Figure 2:**
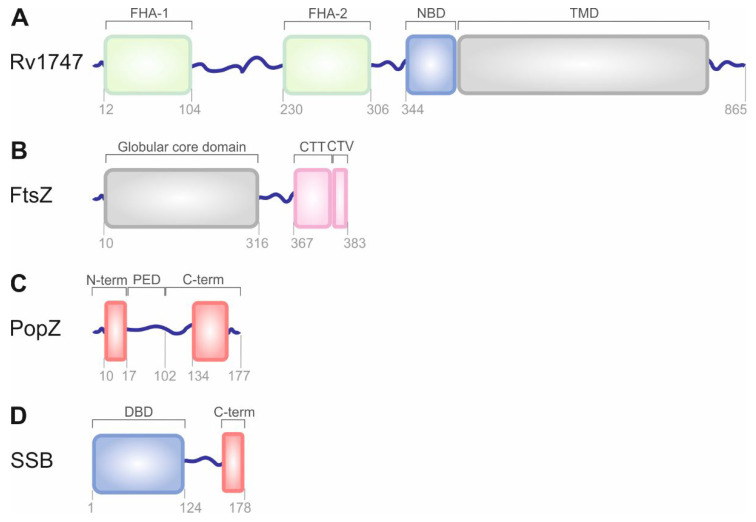
The structural organization of bacterial proteins with propensity for LLPS. (**A**) Architecture of the ABC transporter Rv1747. Rv1747 is composed of a cytoplasmic regulatory module containing the forkhead-associated (FHA-1 and FHA-2) domains joined by an ID linker, cytoplasmic nucleotide-binding domain (NBD), and a helical transmembrane domain (TMD) through which substrate is transported. (**B**) Domain organization of FtsZ protein. FtsZ contains 10 unstructured residues at the N-terminus, a conserved globular core domain containing the GTPase active site, a flexible variable linker of approximately 50 residues, a conserved C-terminal tail (CTT), and a C-terminal variable region of 4 residues (CTV). (**C**) Schematic representation of PopZ structure. PopZ comprises two conserved and mostly α-helical domains: N- and C-terminal (shown in red) and proline-glutamate rich domain (PED) located between them. (**D**) Domain organization of SSB protein. SSB protein molecule contains an N-terminal well-folded domain that is responsible for DNA binding (DBD), LCR, and C-terminal protein–protein interaction region (C-term). Disordered, flexible linkers are shown as blue lines. These regions are often supposed to drive LLPS.

The *Mycobacterium tuberculosis* ATP-binding cassette (ABC) transporter Rv1747 belongs to a large superfamily of multisubunit permeases that transport various molecules across biological membranes [27]. It is a putative exporter of cell wall biosynthesis intermediates that are important for *M. tuberculosis* growth in infected hosts [28]. This integral membrane protein (monomer) is composed of two forkhead-associated (FHA) domains that are separated from each other by an intrinsically disordered linker, a cytoplasmic nucleotide-binding domain, and a transmembrane domain (Figure 2A) [27]. Heinkel et al. showed that the FHA domains have an intrinsic ability to form spherical liquid-like condensates as a function of concentration and phosphorylation (Figure 1), which is probably due to some nonspecific electrostatic interactions between them and the linkers [29]. Well-defined, liquid-like condensates were made spontaneously in vitro when the Rv1747^1–310^ variant of the transporter was phosphorylated by the kinase PknF [29]. Further investigation revealed the ability of non-phosphorylated Rv1747^1–310^ to undergo phase separation, although the process was observed at a significantly higher saturation concentration than that of the PknF-treated protein. Additionally, the process of phase separation was reversed after the addition of the *M. tuberculosis* phosphatase PstP. Interestingly, both enzymes, PknF and PstP, were found to be present in phosphorylated Rv1747^1–310^ droplets. PknF was uniformly distributed throughout the whole volume of the condensate. PstP, on the other hand, was observed as separated spots on the surface between the condensate and the surrounding solution and exhibited no ability to penetrate the condensate [29]. Finally, single-molecule localization microscopy (SMLM) revealed that the endogenous *M. tuberculosis* Rv1747 forms nonuniformly higher-order nanoclusters within the *Mycobacterium* membrane, and these nanoclusters are analogous to eukaryotic nanoclusters [30] as they may change substrate specificities or allosterically regulate the activity, indicating the possibility of nanoclustering a bacterial ABC transporter [29].

### 2.3. Phase Separation of FtsZ and SlmA as Key Players in Bacterial Cell Division

Bacterial cell division is a strictly controlled process that is orchestrated by a macromolecular complex of proteins that affect cytokinesis, the so-called divisome, and more than 35 protein members have been identified thus far; a tubulin-like GTPase called filamenting temperature-sensitive mutant Z (FtsZ) has been particularly focused on. After binding to GTP, FtsZ monomers polymerize into a dynamic ring-like structure called the Z-ring [31], which functions as a scaffold, controls the timing, and defines the future site of cell division. FtsZ is the first protein to localize at the division site. An additional function of the Z-ring is to recruit other cell division proteins to the mid-cell region to produce a new cell wall between the dividing cells [32]. In addition, FtsZ itself may exert cytokinetic forces that lead to cell division [33]. FtsZ is a structural homolog of tubulin [34]. The protein molecule comprises the following distinct functional regions: a poorly conserved N-terminal peptide [34]; a highly conserved globular core region [35]; a mostly disordered C-terminal linker [36]; a short, conserved C-terminal tail [37]; and a C-terminal variable region (Figure 2B) [38]. FtsZ is widespread, and its homologs can also be found in a number of eukaryotes, which might be required for the division of either chloroplasts [39] or mitochondria [40]. Crucial interactions of FtsZ with other divisome proteins are mediated through the two conserved domains. In *Escherichia coli*, the C-terminal tail of the protein binds to negative spatial regulators of FtsZ assembly, such as septum site-determining protein MinC [41], septation ring formation regulator EzrA [42], and nucleoid occlusion factor SlmA [43], as well as positive regulators with cell division proteins FtsA [44], ZipA [45], SepF [46], or ZapC [47] and ZapD [48] among them. The core region of FtsZ has been reported to interact with the modulatory protein SulA [49]. Moreover, all the Z-ring and other components of the divisome remain highly dynamic at the mid-cell both before and during constriction of the cell, which seems to be crucial for efficient and accurate division.

Formation of the FtsZ droplets was initially observed in the presence of SlmA, which itself was bound to DNA, and the process was reversible [50]. SlmA is an important nucleoid occlusion effector that prevents Z-ring formation and cell division over the nucleoid. The protein acts as a DNA-associated cell division inhibitor. It simultaneously binds chromosomal DNA and FtsZ and disrupts the assembly of FtsZ polymers [43]. Using confocal microscopy imaging, Monterroso et al. observed that the binding of SlmA to SlmA-binding sequences (SBS) before Z-ring formation helped to sequester FtsZ within condensates near the cell membrane [50]. The resulting condensates were dynamic and allowed FtsZ, in the presence of GTP, to undergo polymerization into protein fibers. The condensate formation was dependent on the concentration of the crowding agent and on the ionic strength. Further experiments that simultaneously used both proteins, FtsZ and SlmA/SBS, encapsulated within phase-separated microdroplets to mimic intracellular conditions, demonstrated that FtsZ/SlmA/SBS condensates preferred the lipid interface of the droplets, whereas FtsZ fibers localized to DNA-rich regions [50]. Recently, FtsZ was also demonstrated to form condensates in vitro on its own, albeit less efficiently [51], which suggests that the protein has the intrinsic ability to form biomolecular condensates. Eventually, Robles-Ramos et al. reported that FtsZ can form condensates in crowded solutions that mimic the environment of the cytoplasm and when reconstituted in synthetic cytoplasm-like microdroplets. Moreover, the data showed that condensate formation depended on FtsZ being in the GDP-bound state, whereas, after the addition of GTP, reversible condensate-into-filament conversion was observed. The authors noticed that the ability for condensation was retained, although reduced, even after the C-terminal disordered region of FtsZ [51] was deleted, suggesting that FtsZ has an intrinsic ability to form biomolecular droplet phases. FtsZ condensates have still not been reported in vivo.

### 2.4. PopZ Condensates Found in Cell Pole Organization

Pole-organizing protein (PopZ) is a small, acidic protein found in α-proteobacteria and is essential for maintaining bacterial chromosome organization and normal cell division [52]. Holmes et al. showed that ∼75% of *Caulobacter* PopZ is intrinsically disordered (Figure 2C), which, similar to p53 and other hub proteins in eukaryotic signaling networks, seems to be necessary for multiple protein–protein interactions [53]. In contrast, the remaining part of the PopZ molecule is likely to be structured (Figure 2C), which makes it important for assembling the protein molecules into a macromolecular scaffold [54]. PopZ is prone to self-associating into higher-ordered dense clusters that form microdomains at the cell poles, which allow the entry of several proteins but exclude larger macromolecules, such as ribosomes or chromosomal DNA [55]. Recently, fluorescence microscopy analyses revealed that PopZ formed condensates with a spherical morphology in vitro and that IDRs of the protein molecule were necessary and sufficient for condensate formation [56]. The same authors noticed that the phase behavior of PopZ condensates was a function of protein and salt concentration. Furthermore, after heterologous expression of PopZ in *E. coli* cells, it was found that the diffusion of the protein within the condensates in living cells could be modulated by changing environmental conditions that typically affect protein self-association (e.g., pH, osmolarity, crowding agent concentration). Finally, it was shown that phase separation within PopZ condensates was promoted at low levels of ATP and in the presence of 1,6-hexanediol (in a concentration-dependent manner) but was inhibited by lipoic acid [56]. *Caulobacter* divides asymmetrically, and PopZ microdomains localize to both poles but incur some different effects. Therefore, it is attractive to hypothesize that the cytoplasmic MLOs of PopZ may act as hubs that facilitate the integration of different cellular processes at different places.

### 2.5. ParABS System/ParB-parS Clusters Are Formed via Liquid–Liquid Phase Separation

The tripartite ParABS system, which consists of an ATPase protein ParA, a CTPase and DNA-binding dimeric protein ParB, and a centromere-like *parS* site, mediates the chromosome segregation process in the majority of bacterial species [57]. Within this partitioning system, a centromere-like site, *parS*, is commonly found near the origin of replication, where it stays bound by ParB [58]. ParB has, however, the ability to bind not only to high-affinity *parS* sites but also to adjacent nonspecific DNA molecules [59]. Finally, an ATPase with DNA-binding activity, ParA, acts as a motor protein to drive the segregation of the ParB-*parS* complex, along with the attached DNA cargo [60]. The molecular mechanisms underlying the assembly of the partition complex are still not completely understood, especially compared to the mechanisms governing chromosome segregation in eukaryotes. Recently, however, it has been demonstrated that the bacterial partition complex is formed via LLPS. Using single-molecule tracking and superresolution microscopy, Guilhas et al. demonstrated that *parS* and ParB associate in vivo to form nanometer-sized membrane-free spherical condensates (Figure 1) [61] between which ParB molecules are able to diffuse spontaneously within the same cell, as shown using a combination of FRAP and fluorescence lifetime imaging microscopy (FLIM) measurements. Detailed analyses suggested that ParB molecules, in fact, coexist in two, rather than one, well-defined phases. The first and most abundant phase was characterized as a highly condensed, liquid-like state that contains a high concentration of low-mobility ParB dimers. The second phase resembled a diluted gas-like phase with single, high-mobility ParB dimers diffusing on the nucleoid by weak nonspecific DNA interactions [61]. Importantly, interactions of ParB molecules with chromosomal DNA and with themselves were crucial for the observed phase separation, whereas the nucleation of the ParB condensate required interactions between ParB and the *parS* centromeric sequence [61]. Moreover, real-time imaging after ParA degradation revealed that the ATPase activity of ParA prevented the fusion of ParB condensates and was essential for the condensates to become properly positioned inside the cell [61]. Further experiments performed by Babl et al. showed that *Corynebacterium glutamicum* ParB, as well as its orthologs (*Caulobacter crescentus* and *Thermus thermophilus* ParB), also underwent LLPS in vitro upon exposure to synthetic crowders and that these condensates were prone to dissolution after the increase in ionic strength [62]. Subsequently, the authors tested the influence of additives on the ParB phase separation process and demonstrated that the interaction with either *parS* or cytidine triphosphate might exert some stabilizing effects; however, these effects rely on independent mechanisms [62]. All these findings demonstrate the propensity of ParB for phase separation both in vivo and in vitro and indicate that the mechanism of phase separation regulation might be a widespread and common feature, even for distantly related bacteria.

### 2.6. Bacterial RNA Polymerase Can Form Phase-Separated Transcriptional Foci

RNA polymerase (RNAP) is the enzyme responsible for the transcription of RNA. It initiates the process at specific DNA promoter sequences, and its activity is modulated by numerous transcription factors. In contrast to eukaryotes, which contain three distinct types of enzymes, in bacteria, a single type of RNAP synthesizes different species of RNA. Localization studies in *E. coli* revealed that the core of the enzyme is distributed within the nucleoid and is not present in the cytoplasm [63]. In *Bacillus subtilis* cells, Lewis et al. observed that RNAP can be concentrated in dense foci within the nucleoid (Figure 1) [64]. Detailed studies utilizing fluorescence microscopy revealed that the clustering pattern of bacterial RNAP is extremely sensitive to growth conditions [65], and superresolution imaging provided evidence that transcriptional clusters contain active RNAP molecules [66]. Ladoucer et al. investigated the molecular bases of RNAP clustering to understand the mechanism of cluster formation, which has been debated for years. During the log phase, bacterial DNA is compacted into a filamentous structure [67]. Since RNAP binds to DNA, it was proposed that its clustering is the result of the change in the nucleoid morphology. To determine the type of interaction between macromolecules within these transcriptional RNAP clusters, Ladoucer et al. treated cells with the aliphatic alcohol 1,6-hexanediol. This is a compound that is commonly used in LLPS studies [8]; 1,6-hexanediol disrupts weak hydrophobic interactions but does not interfere with specific or nonspecific protein–DNA interactions [68,69]. Ladoucer et al. found that RNAP-containing clusters dissolved rapidly and reformed once the alcohol was washed away, indicating that the integrity of the foci was not maintained by direct RNAP–DNA interactions. The molecules within the RNAP clusters are dynamic and diffuse rapidly, indicating that the interactions between molecules are weak and transient. Eukaryotic RNAP II was also shown to concentrate into different clusters through LLPS [70,71]. It was shown that the phosphorylation status of the C-terminal domain of eukaryotic polymerase determines the positioning of the enzyme during gene expression. Once the domain is unmodified, it is partitioned into a transcription initiation complex, but, upon phosphorylation by cyclin-dependent kinases, the enzyme is located in splicing-related condensates [72]. RNAP II was found to be localized in transcriptional condensates along with other proteins that are essential for initiating transcription [73]. Similarly, Ladoucer et al. found that the other proteins accompany the clustering of prokaryotic RNAP. The authors also tested several proteins and found that some can undergo spontaneous LLPS in vitro and play a role in cluster nucleation. Among them, NusA, which is a transcription elongation factor, plays a dominant role [74].

Importantly, since ribosomes drive protein synthesis, their number and rate of function determine the accumulation of cytoplasmic mass. It is assumed that during intensive bacterial growth on nutrient-rich media, up to 90% of transcribed RNA is rRNA [75]. Recently, eukaryotic RNAP I, of which the main function is the transcription of the rRNA gene, was found to be involved in the formation of condensates through self-association, together with other nucleolar components [76]. Therefore, in rapidly growing cells, RNAP transcriptional clusters might be considered functional analogs of eukaryotic nucleoli [77], the first MLO organelles identified as a liquid condensate that was formed through LLPS [78].

### 2.7. Dps Complexes Are Selectively Permeable and Exhibit Features Typical of Condensates That Are Formed via LLPS

In response to environmental changes, bacterial cells change the pattern of transcribed genes. As mentioned above, once *E. coli* grows in a nutrient-rich medium, most gene expression is linked to rRNA transcription [75], and RNAP forms liquid transcriptional clusters. RNAP changes dramatically when *E. coli* is grown in nutrition-poor media. In this situation, RNAP foci are dispersed [64,65], and transcription is shifted to the synthesis of mRNA. Studies of the protein expression pattern of starved bacterial cells revealed that in the case of a severe lack of nutrients, *E. coli* produces predominantly one type of protein—that is, an oligomeric DNA-binding protein from starved cells (Dps) [79]. This protein functions as a pleiotropic factor, in which the major role is protecting the nucleoid during stress conditions [80]. Upon discovery, the protein was purified, and further in vitro analyses revealed that its binding was nonspecific to the sequence, size, and topology of DNA [79]. Dps has no known canonical nucleic acid-binding motifs and shows a lower affinity for DNA than that of the other nucleoid-associated proteins [63]. Moreover, its inner and outer surfaces were found to be mostly electronegative [81]; thus, its mode of action remained unsolved for years. Dps binding of DNA results in the formation of crystalline DNA arrays [82], and it has been shown that nucleoid condensation is essential for the bacterial stress response [83]. Janissen et al. examined the influence of Dps on the activity of RNAP during a stationary phase. Using a strain of bacteria that lacks Dps expression, they found that Dps does not influence mRNA synthesis and that protein expression is only mildly altered [84]. Since Dps-DNA complexes are dense and compact [85], it was unclear if and how RNAP can perpetrate the nucleoid and actively transcribe DNA at appropriate sites. In elegant studies by Janissen et al., it was found that Dps condensates are permeable for RNAP but not for other DNA-interacting proteins. According to researchers, the explanation for this peculiar behavior lies in the fact that Dps can drive LLPS, so its condensates have a liquid nature (Figure 1) [84]. In fact, Dps-DNA compaction has been shown to be metastable and can be influenced by physicochemical factors, such as salt, pH, or molecular crowders [86]. According to Janissen et al., Dps can form liquid condensates via their disordered N-terminal domain. RNAP can enter the Dps condensate as a client molecule. Therefore, transcriptional flexibility and the transcriptional response are maintained. As a result, under stress conditions, the bacterial nucleoid is protected from damage in dense condensates, but at the same time, the transcription of needed genes can be continued [84].

### 2.8. Bacterial Cells Store a Pool of SSB in Phase-Separated Condensates

Single-stranded (ss) DNA binding (SSB) proteins are highly conserved, pleiotropic regulatory proteins that are identified in all domains of life. Their main function is to protect ssDNA from chemical and proteolytic attack and from the formation of secondary structures that can inhibit RNAP [87]. SSB proteins are also involved in other aspects of DNA metabolism [88,89]. Studies by Harami et al. showed that at ionic strengths that correspond to the assumed concentration of anions and cations within the bacterial cells, the SSB protein forms regular spherical droplets that resemble liquid condensates formed by LLPS [90]. The SSB protein is multidomain and partly disordered (Figure 2D) [87,91]. In vitro studies on deletion mutants revealed that the disordered linker of the SSB protein can form multivalent homotypic interactions that drive LLPS [90]. In *E. coli*, the LCR of the SSB protein is rich in glycine residues. They are present as a series of short linear clusters. These glycine-rich clusters are expected to be key molecular drivers of SSB LLPS [92]. Furthermore, interactions between the disordered C-terminal part of the protein and its globular N-terminal part enhance the process [90]. Currently, it is well known that LLPS can be driven by the interaction between proteins and nucleic acids, mainly single-stranded RNA or DNA [93]. The SSB protein from *E. coli* is a unique example of a protein that can undergo LLPS, but the process is inhibited by nucleic acid binding. Harami et al. showed that ssDNA can penetrate condensates formed by SSB proteins and diffuse freely within them. ssDNA competes with the disordered linker for binding to the N-terminal domain. Once ssDNA accumulates in a cell, the condensates formed by SSB protein dissolve, and the N-terminal part of the protein interacts with nucleic acids. In this elegant way, ssDNA governs the formation of SSB protein condensates and regulates the main function of these proteins, which protects ssDNA from breakage. The observations of [90] suggest that LLPS helps to store the SSB pool at the inner membrane. Since the formation of liquid condensate via LLPS is reversible, the protein can easily be recruited to the site of breakage [90].

### 2.9. LLPS May Determine the Cellular Localization of mRNA

The LLPS-based concept of cellular organization may help to clarify another very important aspect of bacterial biology—mRNA distribution and its cellular localization. Bacteria are prokaryotic organisms that store their genetic material in the form of nucleoids and are believed to spatiotemporally couple transcription and translation. According to this translation-dependent model of mRNA localization, ribosomes penetrate the nucleoid, bind mRNA near its loci, and start translation while transcription is still proceeding. Currently, a translation-independent model of mRNA localization in different subcellular domains is proposed [94,95]. It was shown that mRNA is not only localized near its loci but can also be clustered along the cell membrane or form puncta near the cell pores [94]. Certain mRNAs contain specific *cis*-acting sequences localized in a fragment coding for the transmembrane region of membrane proteins. These sequences are needed to deliver a transcript to the target localization [94,96]. This concept is still enigmatic and requires further examination. However, in connection with the fact that LLPS frequently occurs in proteins that bind RNA [97], it is possible that it also plays a significant role in the translation-independent mode of bacterial mRNA localization. However, this is speculative and requires further in-depth research.

## 3. Phase Transitions of Biomolecules and Material Properties of Bacterial Cytoplasm

In response to environmental factors (e.g., temperature, pH, mechanical stress, component concentration), condensates with liquid-like behavior can undergo phase transitions to form more solid-like forms (e.g., gels, glass, crystals) [98]. These processes have been described for some eukaryotic MLOs and their components. The transition from liquid to solid condensates can be associated with neurodegenerative diseases [99]. However, some condensates (e.g., P bodies, SG granules) and the elements of particular MLOs (e.g., components of the nuclear pore complexes (NPCs) [100], TAR DNA-binding protein 43 (TDP-43) [101]) have tunable phase behavior and can function in both liquid and solid-like states. As in eukaryotic cells, bacterial cells might also have structures that spatially and temporally organize the cytoplasm and exhibit phase behavior to fulfill specific functions (Figure 1).

Inorganic polyphosphate (polyP) is a linear polymer of different numbers of orthophosphate residues that are linked by high-energy phosphoanhydride bonds. This polymer is present in all living organisms, from bacteria to mammals, and possesses distinctive properties. In eukaryotes, polyP stimulates blood clotting, regulates bone mineralization, activates mTOR, and triggers apoptosis [102,103]. In bacteria, polyP acts as an energy and phosphate reservoir. PolyP plays an essential role in responses to stress, cell survival, and cation homeostasis [104,105]. Under states of stress and starvation, bacterial polyP forms structures called polyP granules. Racki et al. investigated the formation of polyP granules in *Pseudomonas aeruginosa* after nitrogen starvation [106]. Granule synthesis was necessary to exit the cell cycle during starvation, and the structures were spatially organized within the nucleoid region. It was proposed that these granules may serve as a microenvironment that compartmentalizes specific enzymatic activity.

The maturation of polyP granules was also observed during starvation. The polyP granules seem to be dynamic structures, as they decrease in number yet increase in size after nucleation (Figure 1). In vitro studies of polyP indicated that it can exhibit diverse biophysical properties on its own, including liquid droplet properties to more solid-like properties (hydrogels, amorphous glasses, and crystals) [103]. The material properties of polyP are impacted by its length. However, it is not known if such a broad spectrum of polyP exists in bacterial cells and how it influences polyP granules. The polymeric nature of polyP, its structural flexibility, and its chemical stability enable it to serve as a scaffold for different biomolecules [104]. Cremers et al. demonstrated that in uropathogenic *E. coli*, polyP serves as a scaffold for amyloidogenic proteins, such as CsgA [107]. PolyP stimulates amyloid-dependent biofilm formation in bacteria by accelerating the transition of amyloid proteins to their fibril-forming β-sheet conformation (called curli). This mechanism explains the stimulatory effects of polyP on the formation of bacterial biofilms. Components of polyP granules might also induce interactions that help to organize the nucleoid during starvation. Wang et al. showed that polyP can control the phase behavior of proteins through electrostatic interactions [108]. In *E. coli*, polyP serves as a protein chaperone during oxidative stress [109]. It stabilizes unfolded proteins by preventing unfolding and irreversible aggregation. The interplay of polyP with other biomolecules may be one of the unifying principles through which polyP achieves its diverse functions.

An interesting example is how the changes in the cellular environment of bacteria might induce phase transitions to form bacterial ribonucleoprotein (BR) bodies, which can be more solid-like biomacromolecular assemblies. They are responsible for RNA decay. These RNA degradosomes contain nucleases (RNases) and RNA decay-associated proteins (RNA helicases, metabolic enzymes) [110]. The content of BR bodies varies significantly across bacteria. The major bacterial mRNA decay nuclease is RNase E [111]. *C. crescentus* RNase E was the first bacterial protein identified that forms LLPS condensates both in vivo and in vitro [111]. Al-Husini et al. also showed that BR bodies dynamically assemble in the presence of RNA and disassemble upon RNA cleavage both in vitro and in vivo [111]. BR bodies share similarities with eukaryotic MLOs that are connected with RNA metabolism, such as P bodies and stress granules [112,113]. The appearance of BR bodies can be strongly induced upon a variety of cell stresses and changes in growth conditions. Al-Husini et al. observed an increased intensity of BR bodies, which was caused by the localization of most of the RNase E into BR bodies upon the addition of cell stress [111]. The increase in BR body intensity was correlated with a decrease in mRNA decay [114]. Muthunayake et al. suggested that the slowdown in metabolic activity and ATP depletion may cause liquid BR bodies to transition to a gel or solid state through decreased ATP hydrolysis by RNA helicases [110]. This might be a new functional aspect of BR bodies. Similar to eukaryotic MLOs (P bodies, stress granules), bacterial BR bodies can alter their function from stimulating mRNA decay to mRNA storage.

Phase transitions in the bacterial cytoplasm may have functions other than storing only particular components. The formation of crystalline assemblies may provide an efficient means for protecting genetic material. As mentioned earlier, *E. coli* Dps have a propensity to form ordered crystalline arrays that enable DNA to be compacted into a dense phase and help the bacteria to survive over a diverse range of stress conditions [82,85]. This could indicate the presence of either a solid or a liquid crystal phase. Janissen et al. proposed that Dps assemblies may retain some features of a fluid, as they showed similar diffusive properties to liquid–liquid phase-separated organelles [84]. It would be interesting to investigate which factors control these phase transitions and whether DNA protection by the formation of crystalline assemblies is generally widespread in prokaryotes.

Phase transition might also be involved in the formation of amyloids. Amyloid peptides/proteins in eukaryotes (e.g., α-synuclein [115], tau [116], and TDP 43 [117]) can undergo LLPS before the formation of amyloid fibrils. However, the exact role of LLPS in amyloid aggregation at the molecular level remains unclear. Amyloids are classically associated with human diseases. They can also have important physiological functions [118,119]. Bacteria have been shown to use fibril formation for their mechanical properties and to promote host invasion processes. Recently, functional amyloids have been discovered in *Staphylococcus aureus* [120]. Tayeb-Fligelman et al. showed that peptide phenol-soluble modulin α3 (PSMα3) formed elongated fibrils that were characteristic of eukaryotic cross-β fibrils but differed in their secondary structure elements [120]. Detailed analysis revealed a distinctive “cross-α” amyloid-like architecture. These functional amyloids play an important role in the pathogenicity of *S. aureus*. The connection between LLPS and amyloid formation by PSMα3 needs further investigation.

Proteinaceous infectious particles (prions) represent a particular subclass of amyloids and were once thought to occur only in eukaryotic cells. Yuan and Hochschild showed that the transcription termination protein Rho from *Clostridium botulinum* can adopt alternative conformations [121]. They observed that the protein forms amyloid aggregates and functions as a prion. The conversion to the prion conformation influenced Rho’s function, causing decreased Rho activity. It would be very interesting to verify whether Rho has the ability to undergo LLPS and whether the phase transition of Rho precedes the formation of the prion form.

Phase transition might also be involved in the formation of rigid amyloid-like aggregates called inclusion bodies (IBs) [122,123]. IBs occur naturally in bacteria [124], but they are especially common during high-level expression of heterologous proteins [125,126]. The relative abundance of overexpressed recombinant proteins in IBs varies according to the amino acid sequence [127,128] and depends on the conditions of bacterial culture and gene expression [129,130]. IB formation was formerly considered to occur passively by the irretrievable deposition of partially folded intermediates [131]. However, IBs are not only undesired bacterial products (unspecific precipitation of unfolded chains) but can also contain functional protein particles [132,133]. An increasing body of evidence indicates that bacterial aggregation is a rather selective process that is modulated by the protein sequence and conformation [122]. The formation of IBs might be mediated by the cellular machinery (e.g., chaperones) [124]. It has been suggested that IBs are dynamic structures that are formed by an unbalanced equilibrium between partially folded expressed proteins, which aggregate through noncovalent hydrophobic forces or ionic interactions or a combination of both, and the soluble proteins of *E. coli* [131,134]. These stable protein deposits can be stored until the cell system is able to further process them.

The adaptation of bacterial cells to internal and external changes might require phase transitions that not only occur with particular components or condensates but also with the whole cytoplasm. Parry et al. showed that the bacterial cytoplasm behaves differently from a simple viscous fluid [135]. The dynamics and fluidity of the bacterial cytoplasm were dramatically altered through modulating the cellular metabolism by environmental stresses. Depending on the metabolism, component sizes, and interactions, the bacterial cytoplasm displayed properties that were characteristic of glass-forming liquids and could solidify to resemble soft glass [135]. The lower metabolic activity inhibited the motion of cytoplasmic components, and the bacterial cytoplasm underwent a phase transition from a liquid to a glass-like state. Additionally, component mobility was restricted in a size-dependent manner. Components smaller than 30–40 nm were not affected by metabolism-dependent motion. Changes in metabolic activity enhanced the motion of cytoplasmic components by “fluidizing” the cytoplasm. It is incredible how fluctuations in the environment can modulate cytoplasmic dynamics by affecting metabolism. Increasing evidence in eukaryotes and prokaryotes indicates that phase transitions can provide an adaptive response to small changes in the cellular environment.

There are common physical properties of bacterial and eukaryotic proteins (e.g., IDRs, LCRs) that drive the formation of biomacromolecular condensates and their further phase transitions [136]. The formation of such structures inside different types of cells can help cells to respond to different environmental factors and regulate vital functions and can help to maintain cell fitness. Bacterial cells can no longer be considered amorphous “bags of enzymes” [111]. The diversity of biomolecules allows condensates to evolve very quickly so that bacteria can better adapt to their diverse niches. Nevertheless, there are many challenges when investigating phase transitions in bacterial cells, especially in determining the difference between liquid condensates and more solid-like structures. Both assemblies are spherical, and their size depends on the concentration of the components and whether they are combined to form larger assemblies. However, work on the development of new experimental techniques is ongoing.

## 4. Methods to Study Phase-Separated Condensates in Prokaryotic Systems

The significance of LLSP in biological systems has been studied for more than a decade. Since pioneering work demonstrated that eukaryotic MLOs have liquid properties [78,137], a concise methodology has been used to identify molecules that can drive phase separation and to study phase-separated cellular condensates [8,138,139]. The LLPS phenomenon, as an organizer of living matter, has been studied mainly with the consideration of eukaryotic proteins. For in vitro studies, the investigated proteins are purified to homogeneity and analyzed using spectroscopic and microscopy methods [140]. The set of recommended in vitro experiments can easily be applied to studies on prokaryotic proteins. Discrepancies begin to appear in the case of in-cell experiments, which are mainly based on direct microscopic observation. Bacterial cells are simply too small to be subjected to direct microscopic observations of their condensates. The average size of bacterial cells ranges from a few up to several micrometers [141]. Consequently, the typical bacterial phase-separated condensates may be an order of magnitude smaller, so they are beyond the limit on the spatial resolution of light microscopy. For this reason, prokaryotic condensates may not be visible when subjected to direct microscopy observations. 

Recent techniques generally known as superresolution microscopy aid studies on bacterial cell biology. In particular, in cell studies of LLPS and liquid condensates in prokaryotic systems, they are often studied by means of superresolution imaging and single-molecule trafficking methods [142]. Single-molecule localization microscopy (SMLM) provides an extremely high resolution and allows for quantitative analysis. Its lateral spatial resolution ranges from 10 to 30 nm, and its axial resolution is approximately 50 nm [143]. In general, in SMLM techniques, which include stochastic optical reconstruction microscopy (STORM), photoactivated localization microscopy (PALM), and their various modifications, individual fluorophores are activated, imaged, and bleached. The signal, from numerous repeating cycles, is collected and a picture coming from all activated fluorophores is developed. While PALM often uses genetically modified proteins, STORM usually utilizes organic dyes and antibodies. At present, SMLM is a powerful technique but faces a number of limitations. It requires appropriate sample preparation. The fixation step must be optimized to avoid disturbance of fine cellular structures. It has low throughput and is susceptible to reconstruction artefacts, mainly due to a difficulties in avoiding sample drift and the overlapping of emitting fluorophores [142,143]. Nevertheless, this approach allows for the precise determination of molecular trajectories and provides dynamic information on molecules in a condensate [144,145]. For this reason, advanced diffraction-unlimited microscopy and single-cell trafficking have been successfully applied to study phase-separated condensates in prokaryotic cells [146]. It can be expected that with the development of such observation techniques, the LLPS phenomena will be explored in prokaryotic cells, and in the near future, there will be more fascinating examples of this process in bacterial cells and its importance for the organization of the bacterial interior.

## 5. In Silico Analyses of Bacterial Proteins with Propensity for LLPS and Condensate Formation

Bioinformatic predictors are becoming increasingly powerful tools for the preliminary investigation of amino acid sequences to indicate the propensity of proteins to undergo LLPS. In bacterial cells, due to their small size, it is particularly difficult to study the LLPS phenomenon. Thus, predictor-based preliminary analyses can be useful. Proteins that undergo LLPS very often contain IDRs and LCRs [6,147]. This is why disorder predictors, such as IUPred [148], MobiDB [149], and PONDR [150], are often combined with LLPS predictors. Such complex analysis can indirectly help to identify proteins with a propensity for LLPS by providing additional information on their structure. To date, numerous LLPS prediction tools based on different algorithms have been designed. One of the first was catGRANULE [151], an algorithm initially developed for the prediction of granule formation in yeast. It is based on a method of differentiating proteins that are located in granules according to the presence of IDRs, their length, their ability to bind nucleic acids, and the rich content of specific amino acid residues (e.g., R, G, F residues), which are characteristic of granule-forming proteins [152,153]. In 2017, PScore [154] was developed. This tool is based on the analysis of π-π interactions. The set of available predictors also includes PSPer [155], PSPredictor [156], FuzDrop [157,158], PLAAC [159], and PSAP [160]. The algorithms mentioned therein operate on different principles. They determine the propensity for LLPS directly from amino acid sequences (e.g., catGRANULE, described above) or are based on a comparison of the analyzed sequences with data deposited in databases (e.g., PSPredictor is a sequence-based tool integrated with the LLPSDB database).

The bioinformatic analysis of previously discussed proteins, for which LLPS propensity has been experimentally demonstrated, was conducted. It allowed us to evaluate the consistency of the obtained predictions with the experimental data as well as their utility. To date, analyses of this nature have not been performed for these proteins. In addition, we analyzed selected proteins to classify whether they exhibit features of the proteins involved in the formation of MLOs. For this purpose, we used the sequences of these proteins from the UniProt database as inputs to determine the presence of IDRs (PONDR http://www.pondr.com/, accessed on 5 May 2022) and to predict the propensity of the examined proteins to undergo LLPS (FuzDrop https://fuzdrop.bio.unipd.it/predictor, accessed on 5 May 2022). Despite the multitude of available LLPS predictors, the FuzDrop tool was chosen. It provides data such as residue-based droplet-promoting probabilities (pDPs), probability of forming a droplet state through liquid–liquid phase separation (pLLPS), and the presence of probable droplet-promoting regions (pDPRs). Based on these results, it is possible to assign proteins with a role in MLO formation. According to FuzDrop, scaffold proteins are characterized by the presence of pDPRs and pLLPS values above 0.6. If the sequence contains pDPRs but pLLPS is below the threshold value of 0.6, the protein is classified as a client. Additionally, the presence of a long pDPR is typical of scaffolds rather than clients. Proteins lacking a pDPR, regardless of the pLLPS value, are unrelated to the MLO formation process. In eukaryotes, LLPS might occur before the formation of amyloid fibrils [115,116,117]. In bacteria, the link between these processes remains unknown. We aimed to analyze whether these processes are linked at least at the amino acid sequence level. The FuzDrop also predicts the propensity to form solid condensates by determining the presence of aggregation hot spots. For these regions, the pDP value is typically above 0.6.

**Figure 3:**
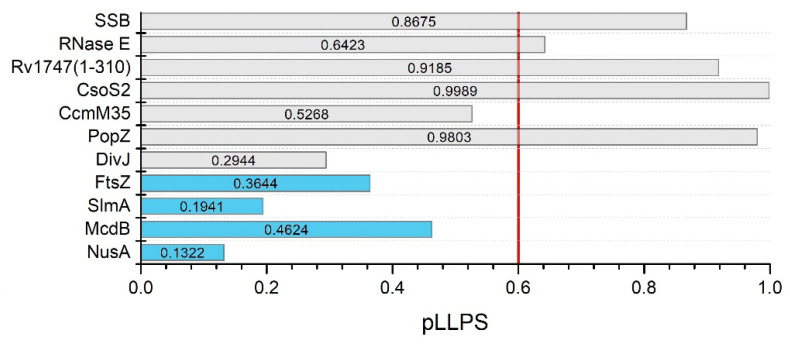
In silico analysis of LLPS-related proteins. Summary graph of the pLLPS values obtained with FuzDrop for SSB (P0AGE0), RNase E (A0A0H3CAR6), Rv1747^1–310^ (O65934), CsoS2 (O85041), CcmM35 (Q03513-2), PopZ (Q9A8N4), DivJ (Q03228), FtsZ (P0A9A6), SlmA (P0A9A6), McdB (Q8GJM6), and NusA (P0AFF6). The gray bars show pLLPS values of proteins for which the in silico data were consistent with the experimental data, the blue bars show pLLPS values for proteins for which the in silico data were found to be inconsistent with the experimental data. The red line is the threshold of 0.6 pLLPS values.

The first analyzed group included the SSB protein, RNase E, Rv1747^1–310^, CsoS2, CcmM35, PopZ, and DivJ. For these proteins, in silico analyses have been shown to be the most consistent with experimental results. Our analyses showed that these proteins represented both groups—potential scaffolds (SSB protein, RNase E, Rv1747^1–310^, CsoS2, PopZ) and clients (CcmM35 and DivJ). The first example of a protein classified as a scaffold is the SSB protein. Harami et al. showed that it has IDRs with a propensity for LLPS [90]. Our analysis indicated that this amino acid sequence contains numerous disordered regions, the longest of which includes the C-terminal domain (Figure S1A). Importantly, FuzDrop showed that this domain contains pDPRs (Figure S1B), which, combined with a pLLPS value above 0.6 (0.8675; Figure 3), classifies it as a scaffold protein in the formation of clusters, allowing a pool of SSBs to be stored at the inner cell membrane [90]. Another example of scaffolds is RNase E, the main component of BR bodies [161]. *C. crescentus* RNase E was the first bacterial protein identified that forms LLPS condensates both in vivo and in vitro, in which the C-terminal intrinsically disordered domain (Figure S2A) provides a scaffold that interacts with RNA at multiple sites [111]. The FuzDrop analysis showed a pattern that was characteristic for scaffolds; the sequence has two pDPRs, including one longer pDRP that covers almost half of the sequence from the C-terminal sequence (Figure S2B), which is necessary for LLPS, and this result is consistent with the observations of Al-Husini et al. [111]. Additionally, there is a shorter pDPR near the N-terminal domain that overlaps with the IDR. The FuzDrop pLLPS value was above the 0.6 threshold (0.6864; Figure 3), which, when compared with previous data, classifies this protein as a scaffold. This is of high probability, as RNase E has been shown to recruit client degradosome proteins to BR bodies [161]. Analogous analysis was performed for the N-terminal domain of Rv1747, which has been shown to undergo phase separations at high concentrations and in a phosphorylation-dependent manner. The protein comprises two FHA domains linked by an IDR [29]. Analysis of the N-terminal domain of Rv1747 (amino acids 1–310) showed that there is a pDPR in the middle of the sequence, which, by its position, corresponds to the disordered linker (Figure S3A,B). The pLLPS score was 0.9185 (Figure 3), indicating that Rv1747^1–310^ can be classified as a scaffold. Furthermore, we also analyzed full-length Rv1747. Despite the presence of three pDPRs (data not shown), full-length Rv1747 did not indicate LLPS propensity. The FuzDrop pLLPS score was slightly below the threshold (0.5716) and indicated that full-length Rv1747 should act as a client. It would be valuable to test this hypothesis experimentally. The results of this analysis lead us to the question of whether assemblies formed by full-length Rv1747 are formed by LLPS or whether some other, unknown mechanism is responsible for this process. An additional example of a potential scaffold is CsoS2, which is the major component of the *H. neapolitanus* α-carboxysome. It is known to be responsible for the formation of condensates with RuBisCo α [22]. It is believed that CsoS2 is the scaffold and initiates carboxysome formation [162]. The sequence of CsoS2 is a repetitive IDP [162,163]. According to PONDR, its overall degree of disorder was 69.51% (Figure S4A). FuzDrop analysis revealed the presence of numerous pDPRs that overlapped with the occurrence of IDRs (Figure S4B). The pLLPS value for the sequence of this protein is well above the threshold (0.9989; Figure 3). These results allowed us to classify CsoS2 as a scaffold, as suggested earlier [22,162]. It is interesting that the propensity of CsoS2 to undergo LLPS seems to be dependent on the entire sequence, not just the N-terminal domain, which is necessary for interactions with RuBisCo [22]. Interestingly, the β-carboxysome counterpart of the previously discussed CsoS2, CcmM35, has been observed to form condensates only upon interaction with RuBisCo β [23,164]. By analyzing the *S. elongatus* CcmM35 sequence, it can be seen that CcmM35 is highly disordered (Figure S5A). Interestingly, only the two longest IDRs overlapped with the pDPRs (Figure S5B). Despite the presence of pDPRs, the pLLPS value for this sequence is less than 0.6 (0.5268; Figure 3); thus, this protein is classified as a client. Which protein might act as a scaffold during the β-carboxysome formation process remains to be discovered. One more potential scaffold turned out to be PopZ. Recently, Saurabh et al. showed that PopZ forms liquid droplets in vitro [56]. The PopZ sequence analysis using PONDR (Figure S6A) seemed to produce results that were consistent with those of Holmes et al. [53] and Bowman et al. [54]. Analysis using FuzDrop showed that pDPRs overlap with IDRs except for the C-terminal domain (Figure S6B). The presence of long pDPRs and pLLPS scores above 0.6 (0.9803; Figure 3) allowed PopZ to be classified as a potential scaffold protein of the PopZ microdomains. In contrast, DivJ has been investigated and showed no propensity for LLPS in vitro [56]. The analysis indicated that the DivJ sequence contains short disordered fragments that overlap with pDPRs (data not shown), and the pLLPS value is below 0.6 (0.2944; Figure 3). Therefore, DivJ may undergo LLPS and partition into the liquid PopZ microdomain, but the client protein DivJ lacks the ability to spontaneously form liquid condensates. The analyses that we performed allowed us to ascribe the role to the studied proteins in the formation of MLOs, and potential scaffolds and clients were identified. Interestingly, each of the scaffold proteins described thus far is characterized by the presence of aggregation hot spots, as determined by FuzDrop (Figures S1B, S2B, S3B, S4B, S5B and S6B). This is a new, previously unexplored area that needs to be explored further to investigate the role of the above-described proteins in the aggregation process.

The second analyzed group of proteins, for which in silico analyses have been shown to be less consistent with the experimental results, includes FtsZ, SlmA, McdB, and NusA. The first example of the previously mentioned inconsistency is a key cell division protein, FtsZ. FtsZ can form phase-separated condensates in the presence of SlmA [50]. PONDR analysis revealed that both proteins contain IDRs intertwined with ordered regions throughout the sequence (Figure S7A,C). FtsZ contains two short pDPRs that only partially overlap with IDRs (Figure S7B). This information, combined with an FtsZ pLLPS value below 0.6 (0.3644; Figure 3), suggests that FtsZ could be a client protein. It should be mentioned that FtsZ condensates have still not been reported in vivo. SlmA does not contain the pDPRs (Figure S7D), and this result, in combination with a pLLPS value below 0.6 (0.1941; Figure 3), classifies it as a non-LLPS-related protein. The LLPS process also has a link to carboxysome distribution. Once *S. elongatus* carboxysomes are formed, they are distributed along the long axis [165]. The system responsible for their distribution is the McdAB system. It is not clear how McdB interacts with the carboxysome. However, it has been shown that McdB undergoes pH-dependent LLPS in vitro, and this mechanism has been proposed as a way to organize carboxysomes [25]. Not only the McdB of *S. elongatus* but also the McdB of other organisms show features of proteins that undergo LLPS [25,166]. PONDR analysis suggested the presence of a long, disordered N-terminal domain and two shorter IDRs at the C-terminus (Figure S8A). The N-terminal domain is also a pDPR (Figure S8B). According to FuzDrop analysis, the pLLPS value was found to be below the threshold (0.4624; Figure 3), suggesting that McdB plays a role other than that of a scaffold protein. Another example of an inconsistency between the experimental data and the in silico analyses is observed in the in silico analysis of an antitermination factor NusA, the main component of RNAP clusters. The protein interacts directly with DNA-directed RNA polymerase subunit β (RpoC) [74] and other anti-termination factors. Ladouceur et al. have shown that NusA can undergo LLPS in vitro by forming homotypic interactions. It was suggested that NusA might be a scaffold during RNAP cluster formation in *E. coli*. Our analysis showed that the NusA amino acid sequence is significantly disordered (Figure S9A) but lacks pDPRs (Figure S9B) and has a low pLLPS value (0.1322; Figure 3). These results suggest that this protein does not undergo LLPS, either as a scaffold or client. Although the analysis of these proteins shows that they do not have the propensity to form LLPS, an interesting observation is found in the results—each of the analyzed sequences (except SlmA) contains some aggregation hotspots (Figures S7B, S8B, and S9B). Perhaps a phase transition other than LLPS is responsible for their assembly formation. The best way to answer the above questions is to conduct a thorough study.

**Figure 4:**
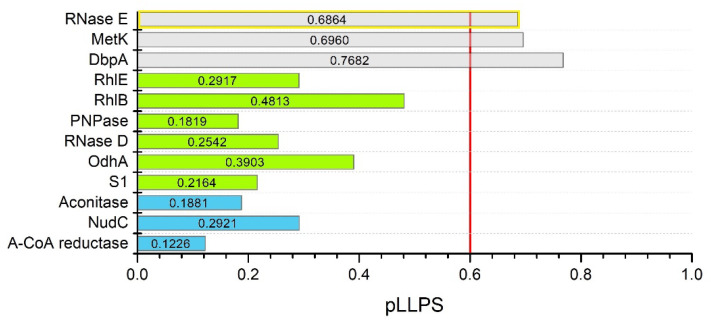
In silico analysis of *C. crescentus* RNase E and degradosome proteins interacting with it. Summary graph of pLLPS values obtained with FuzDrop for RNase E (A0A0H3CAR6), MetK (A0A0H3C5U2), DbpA (A0A0H3C896), RhlE (A0A0H3C5T6), RhlB (A0A0H3C8I9), PNPase (B8GWz0), RNase D (A0A0H3CAA2), OdhA (A0A0H3C4B5), S1 (A0A0H3CCW5), Aconitase (A0A0H3CE29), NudC (A0A0H3C5J2), and acetoacetyl-CoA reductase (A0A0H3C629). Gray bars show pLLPS values for potential scaffold protein sequences (RNase E in yellow box—result confirmed experimentally [111]), green—for clients, blue—for non-LLPS-related proteins. The red line is the threshold of 0.6 pLLPS value.

Eventually, proteins that were not previously studied for LLPS—*C. crescentus* degradosome proteins and *C. botulinum* Rho—were analyzed. As mentioned above, since BR bodies are multicomponent assemblies, we decided to thoroughly analyze selected component proteins of BR bodies to determine what function they might have in the process of MLO formation. Proteins that interact with *C. crescentus* RNase E include polyribonucleotide nucleotidyltransferase (PNPase) [161], ribonuclease D (Rnase D) [167], ATP-dependent RNA helicase RhlB [167], ATP-dependent RNA helicase RhlE [168], DEAD-box RNA helicase-like protein DbpA [168], aconitate hydratase (Aconitase) [161], 2-oxoglutarate dehydrogenase E1 component (OdhA) [168], methionine adenosyltransferase (MetK) [168], NAD^+^ diphosphatase (NudC) [168], acetoacetyl-CoA reductase [168], and S1 ribosomal protein [168]. All these proteins are known to interact with *E. coli* and *C. crescentus* Rnases E [110]. Based on the presence of pDPRs overlapping with IDRs (data not shown) and pLLPS values (Figure 4), the proteins were classified as potential scaffolds (MetK and DbpA), clients (Rhl E, Rhl B, PNPase, Rnase D, OdhA, S1), or non-LLPS-related proteins (aconitase, NudC, acetoacetyl-CoA reductase). These analyses showed that bioinformatics data can be used to find potential new targets for research. Since RNase E has been shown to play a role in degradosome formation as a scaffold, it is curious what role MetK and DbpA might have. In this context, a broader investigation of degradosome proteins for LLPS is needed.

An interesting result was also obtained for *C. botulinum* Rho, which has been observed to form amyloid aggregates and acts as a prion [121]. There is no information about the propensity of Rho to undergo LLPS. In silico analyses showed that the amino acid sequence of Rho contains numerous disordered regions that are located mainly in the middle part of the protein (Figure S10A). Interestingly, Rho has an N-terminal pDPR that does not overlap with any of the IDRs (Figure S10B). This represents some novelty in our analyses. This suggests that IDRs might not only undergo LLPS. The pLLPS value for Rho was found to be 0.3182, which, together with the presence of pDPRs, classifies this protein as client. Little is known about Rho-interacting proteins at this time. Finding a potential scaffold remains a future challenge. In addition, the amino acid sequence of Rho contains aggregation hotspots that are located primarily within pDPR. As Rho has been shown to form amyloids, it represents an interesting system for further studies, and the results could determine whether the LLPS process precedes Rho aggregation.

In summary, sequence analysis does not always reflect the actual properties of biomolecules. Bacterial cells are a complex and dynamic system. Many permanent and transient factors account for the functionality of a given protein, and no predictor can take them all into consideration. Nevertheless, sequence-based predictors serve as very useful tools for preliminary analyses of both the structural and functional biochemical analyses of biomolecules.

## 6. Conclusions

Bacteria are an immanent part of our ecosystem. They degrade waists, neutralize toxins, and produce nutrients. Some affect our lives as symbiotic organisms, while some are pathogenic and may cause life-threatening diseases. Bacteria are also routinely used in laboratory practice, serving as expression systems in the production of recombinant proteins. Therefore, much is focused on understanding aspects of their biology, including molecular changes that accompany adaptation to a new environment, the molecular bases of their stress response, and the resistance that they acquire to toxic compounds, such as antibiotics.

The discoveries of LLPS phenomena in biological systems started a new era in studies concentrated on cell biology. Due to the technical issues and limitations mentioned in this review, bacteria have only recently drawn intense interest in terms of studies on phase separation. Now, we know that some prokaryotic biological processes are in fact controlled by LLPS. In the available literature, there are several fascinating examples of bacterial proteins that have or may have a natural ability to drive phase separation. These examples are acknowledged and discussed in this review. Much has already been understood, yet our bioinformatic analyses indicate that much remains to be revealed, and we anticipate that the library of prokaryotic phase separation proteins will be extended in the near future.

## Data Availability

Not applicable.

## Supplementary Materials

The following supporting information can be downloaded at: https://www.mdpi.com/article/10.3390/biom12070907/s1, Figure S1: In silico analysis of *E. coli* SSB protein sequence; Figure S2: In silico analysis of *C. crescentus* RNase E sequence; Figure S3: In silico analysis of *M. tuberculosis* Rv1747^1–310^ sequence; Figure S4: In silico analysis of *H. neapolitanus* CsoS2 sequence; Figure S5: In silico analysis of *S. elongatus* CcmM35 sequence; Figure S6: In silico analysis of *C. crescentus* PopZ sequence; Figure S7: In silico analyses of *E. coli* FtsZ and SlmA sequences; Figure S8: In silico analysis of *S. elongatus* McdB sequence; Figure S9: In silico analysis of *E. coli* NusA sequence; Figure S10: In silico analysis of *C. botulinum* Rho sequence.
