# Supplementary material for: Getting Closer to Decrypting the Phase Transitions of Bacterial Biomolecules
Source: Biomolecules. 2022 Jun 28;12(7):907. doi: 10.3390/biom12070907 (PMC9312465; doi:10.3390/biom12070907)
Supplement: Supplementary file 1 [file biomolecules-12-00907-s001.zip › biomolecules-1750943-supplementary.pdf]

Supplementary materials

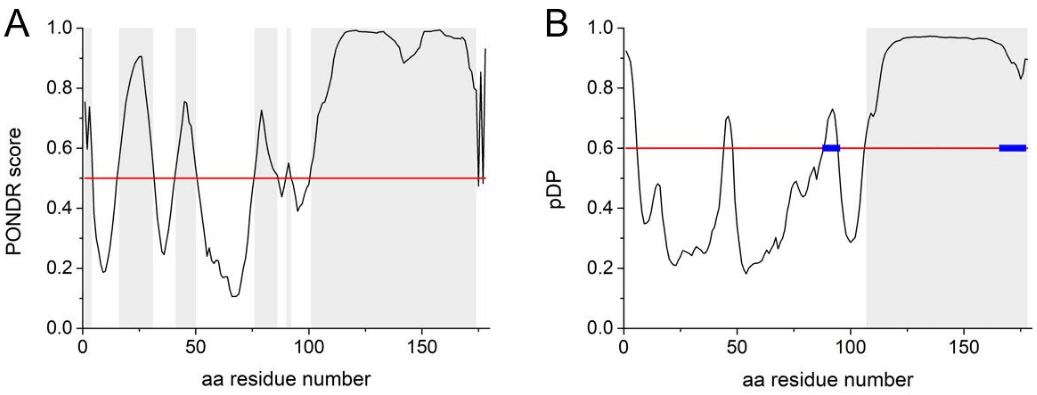

Figure. S1. In silico analysis of *E. coli* SSB protein sequence.

(A) The graph illustrates the prediction of the degree of the disorder in SSB protein (P0AGE0) calculated from its amino acid sequence using PONDR (available at http://www.pondr.com) [1]. A score above 0.5 (red line) indicates a high probability of disorder. The gray areas represent probable disordered segments. **(B)** The graph illustrates FuzDrop analysis (available at https://fuzdrop.bio.unipd.it/predictor) [2,3]. The values above 0.6 (red line) indicate high probability that the protein fragments can drive LLPS. The gray areas represent droplet-promoting regions (DPRs) and the blue bars represent presence of probable aggregation hot-spots.

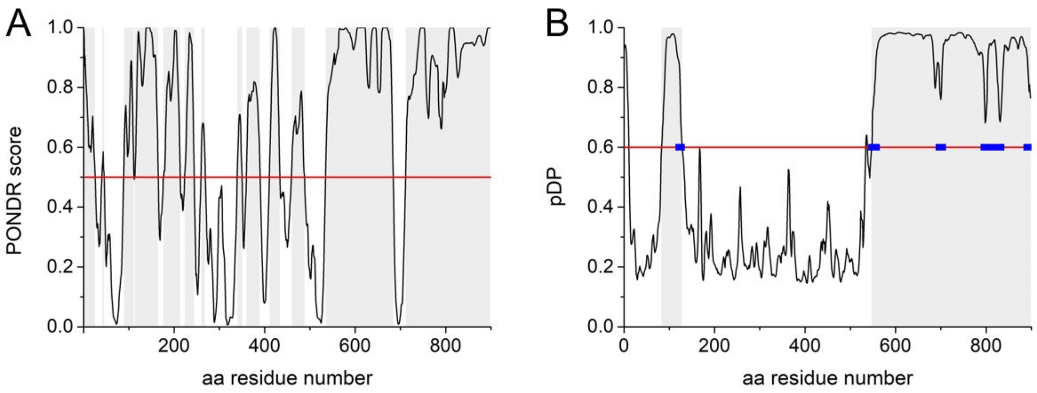

Figure. S2. In silico analysis of *C. crescentus* RNase E sequence.

(A) The graph illustrates the prediction of the degree of the disorder in RNase E (A0A0H3CAR6) calculated from its amino acid sequence using PONDR [1]. A score above 0.5 (red line) indicates a high probability of disorder. The gray areas represent probable disordered segments. **(B)** The graph illustrates FuzDrop analysis [2,3]. The values above 0.6 (red line) indicate high probability that the protein fragments can drive LLPS. The gray areas represent droplet-promoting regions (DPRs) and the blue bars represent presence of probable aggregation hot-spots.

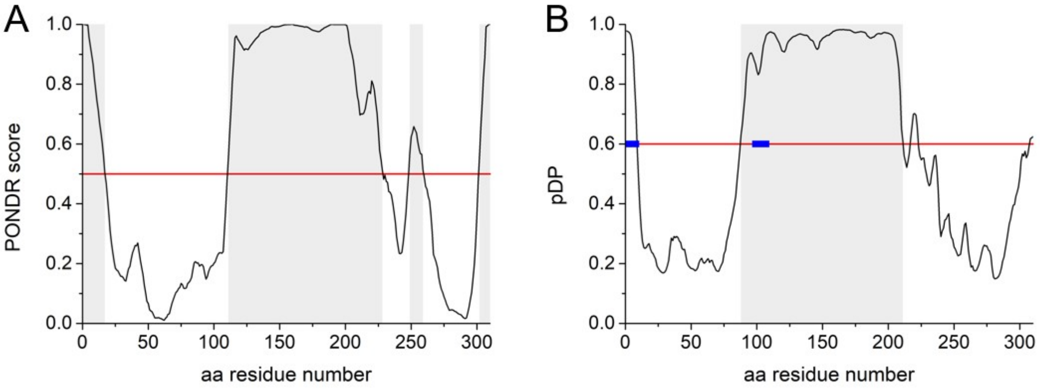

Figure. S3. In silico analysis of *M. tuberculosis* Rv1747$^{1\text{-}310}$ sequence.

(A) The graph illustrates the prediction of the degree of the disorder in Rv1747 (O65934) calculated from its amino acid sequence using PONDR [1]. A score above 0.5 (red line) indicates a high probability of disorder. The gray areas represent probable disordered segments. **(B)** The graph illustrates FuzDrop analysis [2,3]. The values above 0.6 (red line) indicate high probability that the protein fragments can drive LLPS. The gray areas represent droplet-promoting regions (DPRs) and the blue bars represent presence of probable aggregation hot-spots.

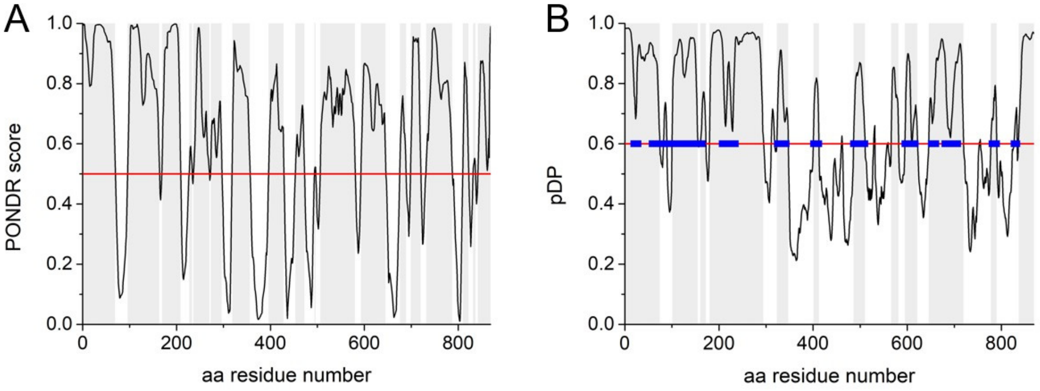

Figure. S4. In silico analysis of *H. neapolitanus* CsoS2 sequence.

(A) The graph illustrates the prediction of the degree of the disorder in CsoS2 (O85041) calculated from its amino acid sequence using PONDR [1]. A score above 0.5 (red line) indicates a high probability of disorder. The gray areas represent probable disordered segments. **(B)** The graph illustrates FuzDrop analysis [2,3]. The values above 0.6 (red line) indicate high probability that the protein fragments can drive LLPS. The gray areas represent droplet-promoting regions (DPRs) and the blue bars represent presence of probable aggregation hot-spots.

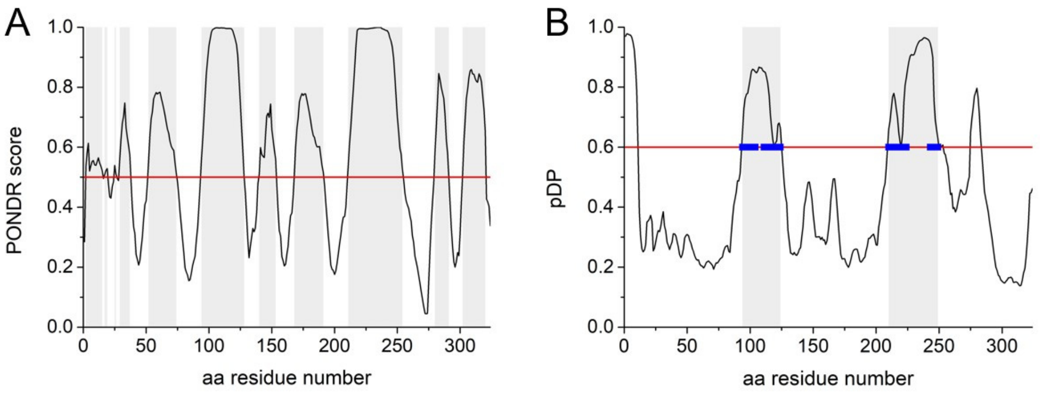

Figure. S5. In silico analysis of *S. elongatus* CcmM35 sequence.

(A) The graph illustrates the prediction of the degree of the disorder in CcmM35 (Q03513-2) calculated from its amino acid sequence using PONDR [1]. A score above 0.5 (red line) indicates a high probability of disorder. The gray areas represent probable disordered segments. **(B)** The graph illustrates FuzDrop analysis [2,3]. The values above 0.6 (red line) indicate high probability that the protein fragments can drive LLPS. The gray areas represent droplet-promoting regions (DPRs) and the blue bars represent presence of probable aggregation hot-spots.

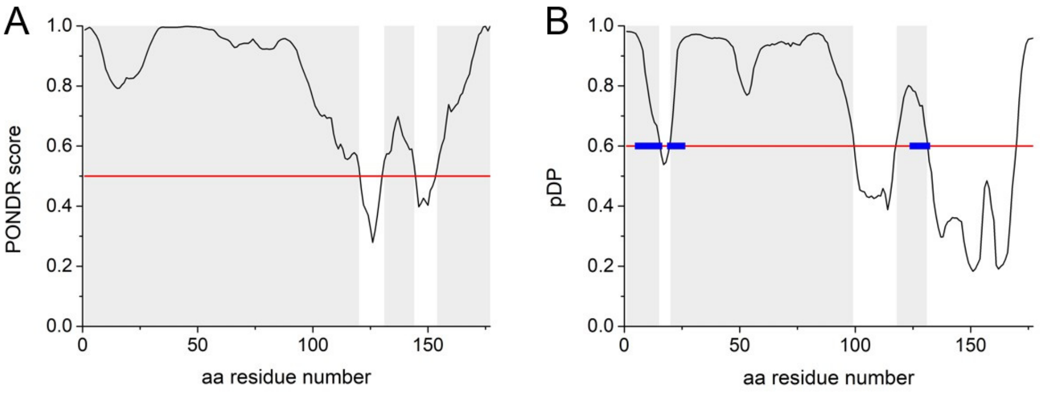

Figure. S6. In silico analysis of *C. crescentus* PopZ sequence.

(A) The graph illustrates the prediction of the degree of the disorder in PopZ (Q9A8N4) calculated from its amino acid sequence using PONDR [1]. A score above 0.5 (red line) indicates a high probability of disorder. The gray areas represent probable disordered segments. **(B)** The graph illustrates FuzDrop analysis [2,3]. The values above 0.6 (red line) indicate high probability that the protein fragments can drive LLPS. The gray areas represent droplet-promoting regions (DPRs) and the blue bars represent presence of probable aggregation hot-spots.

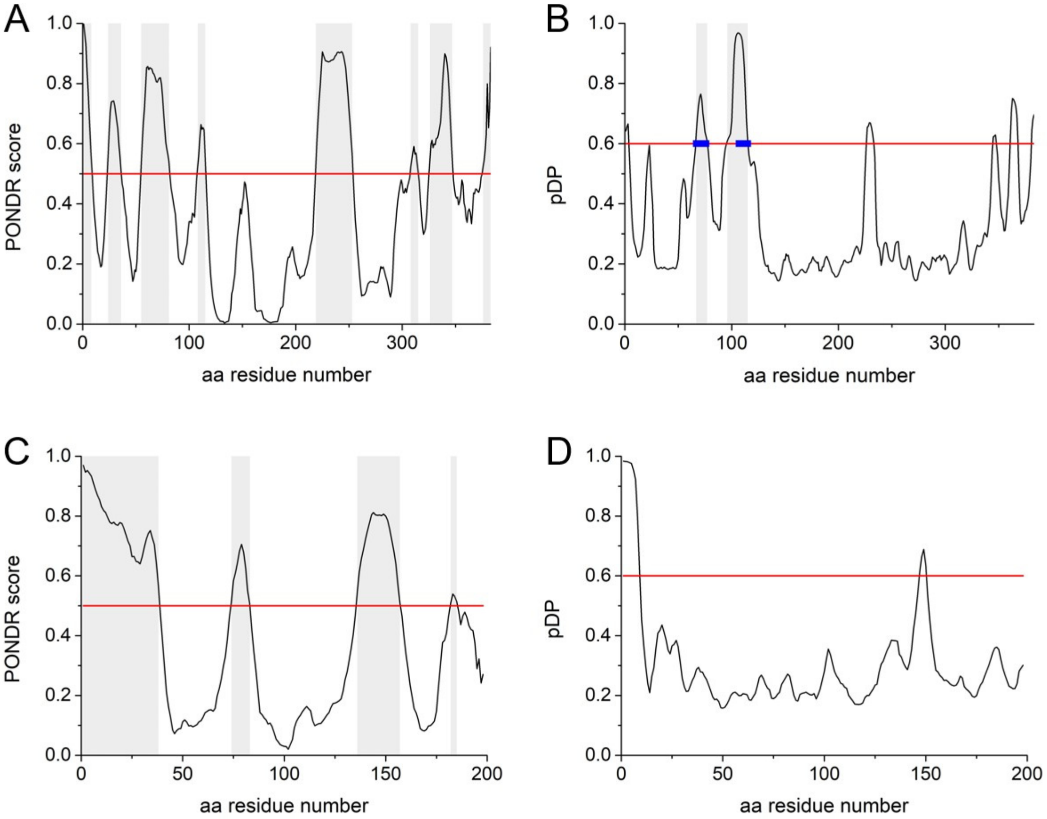

Figure. S7. In silico analyses of *E. coli* FtsZ and SlmA sequences.

(A)(B) In silico analyses of FtsZ (P0A9A6). **(C)(D)** In silico analyses of SlmA (P0C093). **(A)(C)** The graphs illustrate the predictions of the degree of the disorder calculated from the amino acid sequences using PONDR [1]. A score above 0.5 (red line) indicates a high probability of disorder. The gray areas represent probable disordered segments. **(B)(D)** The graphs illustrate FuzDrop analyses [2,3]. The values above 0.6 (red line) indicate high probability that the protein fragments can drive LLPS. The gray areas represent droplet-promoting regions (DPRs) and the blue bars represent presence of probable aggregation hot-spots.

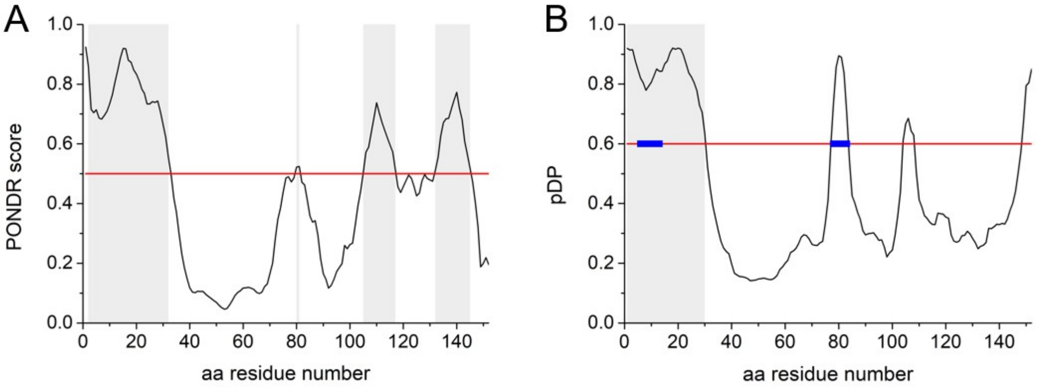

Figure. S8. In silico analysis of *S. elongatus* McdB sequence.

(A) The graph illustrates the prediction of the degree of the disorder in McdB (Q8GJM6) calculated from its amino acid sequence using [1]. A score above 0.5 (red line) indicates a high probability of disorder. The gray areas represent probable disordered segments. **(B)** The graph illustrates FuzDrop analysis [2,3]. The values above 0.6 (red line) indicate high probability that the protein fragments can drive LLPS. The gray areas represent droplet-promoting regions (DPRs) and the blue bars represent presence of probable aggregation hot-spots.

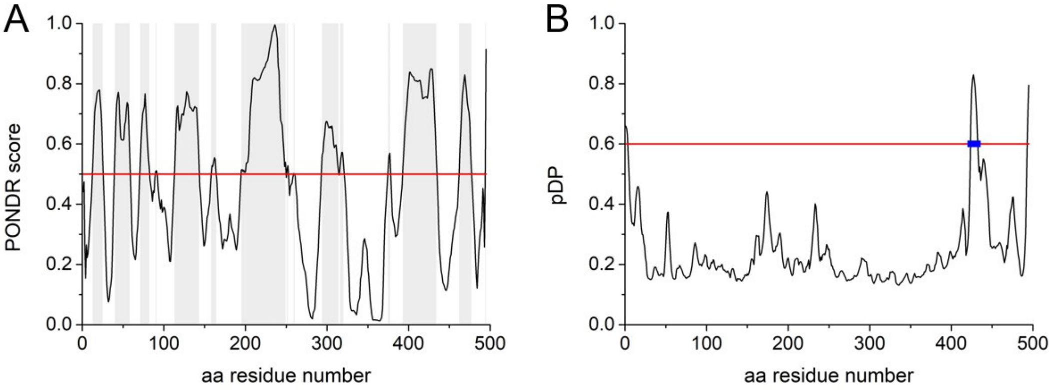

Figure. S9. In silico analysis of *E. coli* NusA sequence.

(A) The graph illustrates the prediction of the degree of the disorder in NusA (P0AFF6) calculated from its amino acid sequence using [1]. A score above 0.5 (red line) indicates a high probability of disorder. The gray areas represent probable disordered segments. **(B)** The graph illustrates FuzDrop analysis [2,3]. The values above 0.6 (red line) indicate high probability that the protein fragments can drive LLPS. The gray areas represent droplet-promoting regions (DPRs) and the blue bars represent presence of probable aggregation hot-spots.

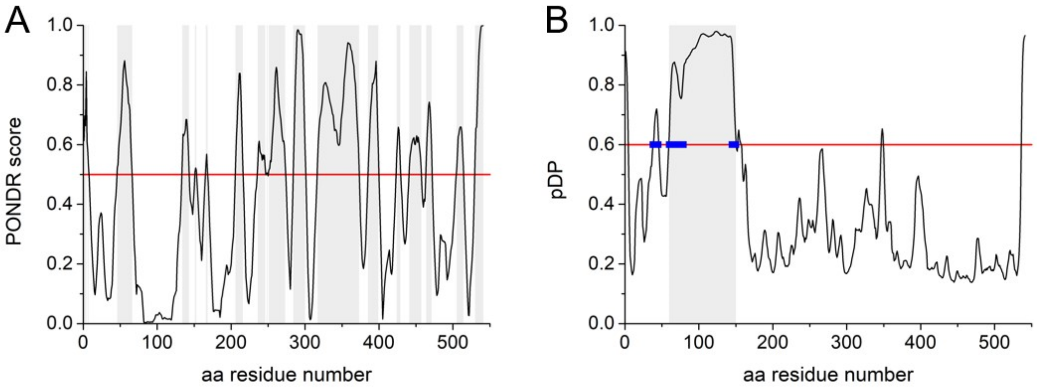

Figure. S10. In silico analysis of *C. botulinum* Rho sequence.

(A) The graph illustrates the prediction of the degree of the disorder in Rho (A0A6B4BFS7) calculated from its amino acid sequence using [1]. A score above 0.5 (red line) indicates a high probability of disorder. The gray areas represent probable disordered segments. **(B)** The graph illustrates FuzDrop analysis [2,3]. The values above 0.6 (red line) indicate high probability that the protein fragments can drive LLPS. The gray areas represent droplet-promoting regions (DPRs) and the blue bars represent presence of probable aggregation hot-spots.

References

1. Romero; Obradovic; Dunker, K. Sequence Data Analysis for Long Disordered Regions Prediction in the Calcineurin Family. *Genome Inform. Ser. Workshop Genome Inform.* **1997**, *8*, 110–124.

2. Hardenberg, M.; Horvath, A.; Ambrus, V.; Fuxreiter, M.; Vendruscolo, M. Widespread occurrence of the droplet state of proteins in the human proteome. *Proc. Natl. Acad. Sci. U. S. A.* **2020**, *117*, 33254–33262, doi:10.1073/PNAS.2007670117.

3. Vendruscolo, M.; Fuxreiter, M. Sequence Determinants of the Aggregation of Proteins Within Condensates Generated by Liquid-liquid Phase Separation. *J. Mol. Biol.* **2022**, *434*, 167201, doi:10.1016/j.jmb.2021.167201.
